# Micronutrient Intakes from Food and Supplements in Australian Adolescents

**DOI:** 10.3390/nu6010342

**Published:** 2014-01-14

**Authors:** Caroline M. Gallagher, Lucinda J. Black, Wendy H. Oddy

**Affiliations:** Telethon Institute for Child Health Research, The University of Western Australia, Perth, Western Australia 6008, Australia; E-Mails: cgallagher@ichr.uwa.edu.au (C.M.G.); lblack@ichr.uwa.edu.au (L.J.B.)

**Keywords:** adolescents, food intake, micronutrients, dietary supplements, Raine Study

## Abstract

Objective: Low micronutrient intakes in adolescents are frequently reported. We assessed micronutrient intakes in adolescents to determine whether supplement use optimises intakes. Methods: Dietary intake was assessed using a food frequency questionnaire in 17 year old participating in the Western Australian Pregnancy Cohort (Raine) Study (*n* = 991). We calculated median daily micronutrient intakes in supplement users and non-users (from food sources only and from food and supplements), along with the percentage of adolescents meeting the Estimated Average Requirements (EAR) or Adequate Intake (AI) where appropriate. Results: Intakes of calcium, magnesium, folate and vitamins D and E from food only were low. Although supplements significantly increased micronutrient intakes in supplement users, more than half of supplement users failed to meet the EAR or AI for some key micronutrients. Compared with non-users, supplement users had higher micronutrient intakes from food sources with the exception of vitamins D and B12 and were more likely to achieve the EAR or AI for many micronutrients from food only. Conclusions: Intakes of some key micronutrients were low in this population, even among supplement users. Those facing the greatest risk of micronutrient deficiencies were less likely to use supplements.

## 1. Introduction

Low intakes of micronutrients, including calcium, folate, magnesium and potassium, have been previously reported in Australian adolescents [1]. Similarly, adolescent diets in Europe and the United States (US) have been associated with low intakes of calcium, vitamin D, iron, folate and zinc [2,3,4,5]. Assessing micronutrient status in adolescents is important due to the contribution of micronutrients to disease prevention [6]. Herbison and colleagues reported that low intake of B vitamins was associated with poor mental health and behaviour in adolescents [7]; calcium and magnesium may play a protective role in type 2 diabetes [8]; and young adults with higher magnesium have a lower risk of developing the metabolic syndrome [9]. Furthermore, adequate calcium and vitamin D levels are essential during adolescence, when approximately 40% of total bone mass is accumulated [10,11].

In order to reliably assess micronutrient intakes, the contribution of nutritional supplements to intake must be taken into account [12]. Nutritional supplement use is increasing in many countries and is popular worldwide in adolescents [5,12,13,14,15,16]. The EPIC study in the United Kingdom (UK) found that the contribution of nutritional supplements to nutrient intakes can be substantial, and miscalculation of nutrient intakes can occur if supplement use is not considered [17]. Therefore, we aimed to assess micronutrient intakes in 17 year old adolescents in Western Australia and to determine whether supplement use optimises micronutrient intakes.

## 2. Experimental Section

### 2.1. Participants

Participants were from the Western Australian Pregnancy Cohort (Raine) Study, which has been described previously [18]. In brief, 2900 pregnant women were recruited through the public antenatal clinic at King Edward Memorial Hospital and nearby private clinics in Perth, Western Australia between May 1981 and November 1991. A total of 2868 children were available for follow-up. The King Edward Memorial Hospital and Princess Margaret Hospital Ethic Committees approved the study protocol. The participant and/or their primary caregiver provided written consent for their participation in the study. In order to increase participation at each follow-up, participants were sent regular newsletters, Christmas cards, birthday cards and received regular updates and results of the study. Participants for the 17 year follow-up were contacted by a research assistant over the telephone. A total of 2168 adolescents were eligible for follow-up at 17 years between July 2006 and June 2009. Of these, 1754 individuals participated and 1009 provided dietary intake data.

### 2.2. Dietary Intake

Dietary intake at the 17 year follow-up was assessed using a self-reported semi-quantitative food frequency questionnaire (FFQ) developed by the Commonwealth Scientific and Industrial Research Organisation (CSIRO) in Adelaide, Australia [19]. This FFQ has been validated for reliability against a 3-day food record in the same cohort [20] and also in adults [21]. From the FFQ we collected information on 212 foods, mixed dishes and beverages, including beverages and snacks popular among adolescents. An overall estimate of the adolescents’ usual dietary intakes in the past year was established using the portion size in standard household measures, and the number of times the food was eaten per day, per week or per month. Participants were asked to record any additional items that were consumed regularly but were not included in the FFQ. All questionnaires were checked by a research nurse and queries were clarified with the adolescent. Seasonal differences were accounted for by asking how often foods were eaten in summer and winter. Food intake data were entered into a database and verified by CSIRO. Estimated daily micronutrient intakes were provided by CSIRO using nutrient composition derived from four sources: the Australian nutrient database (NUTTAB95) [22]; the British Food Composition Tables [23]; the US Department of Agriculture food tables [24]; and manufacturers’ data. Questionnaires were excluded if the daily energy intake reported was implausible (<3000 or >20,000 kJ per day). Micronutrient intakes were calculated for thiamin, riboflavin, niacin, pantothenic acid, pyridoxine, vitamin B12, folate, beta-carotene, vitamins A, C, D, E, calcium, iron, potassium, magnesium, zinc, phosphorus and copper.

### 2.3. Supplement Use

Participants were asked to record any supplements they used over the last twelve months, including brand, name of product, dose and frequency of use. Composition data for supplements were obtained from the product label or directly from the manufacturer. If the frequency of use was less than daily, the nutrient intake was calculated to reflect daily intake over the last twelve months. When there were insufficient data regarding the brand, name, dose or frequency of use, a standardised default was used based on the most common supplement of that type recorded by the participants. Micronutrient intake from supplements was added to the intake from food sources to give a total daily micronutrient intake for supplement users.

### 2.4. Demographic Characteristics

Height was measured using a Holtain Stadiometer to the nearest 0.1 cm. Weight was measured using a Wedderburn Digital Chair Scale to the nearest 100 g. Body Mass Index (BMI) was calculated as weight in kilograms divided by height in metres squared. Underweight, normal weight, overweight and obesity were defined according to age- and sex-specific BMI cut-offs [25,26]. Physical activity was assessed using a self-reported questionnaire, based on exercise outside of school hours per week, where exercise was defined as activity causing breathlessness or sweating (≥4 times per week, 1–3 times per week and <once per week). Television/computer viewing was assessed by the amount of hours watching television or using the computer per day (<2 h per day, 2–4 h per day, >4 h per day). Family income was defined as the gross income before tax and was determined as AUD (per year) <$35,000, $35,001–$70,000 or >$70,001 (average gross salary in 2009 was AUD $63,612 [27]). Maternal education level was indexed by whether the mother had completed 12 years of education or not by the time the child was 8 years old.

### 2.5. Statistical Analysis

Chi-square tests were applied to identify differences in sex, BMI category, maternal education, family income, screen use and physical activity between supplement users and non-users. Median daily micronutrient intakes in males and female supplement users and non-users were calculated from food sources alone and from food and supplements*.* The percentage of males and female supplement users and non-users meeting the Estimated Average Requirement (EAR) or Adequate Intake (AI) was calculated. The EAR is defined as the daily nutrient level estimated to meet the requirements of half the healthy individuals in a particular life stage and gender [28]. Where evidence was insufficient or too conflicting to establish an EAR, an Adequate Intake (AI) was set. The AI is defined as the average daily nutrient intake level based on observed or experimentally-determined approximations or estimates of nutrient intake by a group (or groups) of apparently healthy people that are assumed to be adequate [27].

Since most micronutrient intakes were non-parametrically distributed, Mann Whitney-U tests were applied to investigate differences in male and female intakes from food sources in supplement users and non-users. We applied Wilcoxon signed rank tests to identify differences in micronutrient intakes between male and female supplement users, from food sources only and from food sources plus supplements. Chi-square tests were used to determine differences in the percentage of adolescents achieving the EAR from food sources between supplement users and non-users. We used Statistical Package for Social Science for Windows Rel.20.0.0 (Chicago: SPSSS Inc., Illinois, IL, USA) and defined statistical significance as *p* < 0.05.

## 3. Results

### 3.1. Characteristics

At the 17 year follow-up, 1009 participants provided dietary intake data; however, 18 of these were excluded due to implausible energy intakes (<3000 or >20,000 kJ per day) [29]. Ultimately, dietary intake data were available for 238 supplement users (24%) and 753 non-users. Supplement users were more likely to be physically active than non-users (*p* < 0.05) (Table 1). No other significant differences between supplement users and non-users were observed.

**Table 1:** Characteristics of 17 year old adolescents providing dietary intake data in the Western Australian Pregnancy Cohort (Raine) Study.

		Total Population (*n* = 991)	Supplement Users (*n* = 238)	Non-Users (*n* = 753)	*p* value
		*n* (%)	*n* (%)	*n* (%)	
Sex					0.061
	Male	454 (45.8)	96 (40.3)	358 (47.5)	
	Female	537 (54.2)	142 (59.7)	395 (52.5)	
BMI category					0.165
	Underweight	65 (7.7)	13 (6.2)	52 (8.3)	
	Healthy weight	606 (72.1)	158 (75.2)	448 (71.1)	
	Overweight	108 (12.9)	30 (14.3)	78 (12.4)	
	Obese	61 (7.3)	9 (4.3)	52 (8.3)	
Computer and/or television use					0.273
	<2 h per day	33 (3.6)	11 (4.6)	22 (2.9)	
	2–4 h per day	486 (53.1)	124 (52.1)	362 (48.1)	
	>4 h per day	397 (43.3)	89 (39.8)	308 (40.9)	
Physical activity					
	Once per week or less	186 (19.7)	42 (18.4)	144 (20.2)	0.013 *
	1–3 times per week	512 (54.4)	110 (48.2)	402 (53.4)	
	4+ times per week	244 (25.9)	76 (33.3)	168 (23.5)	
Maternal Education					0.082
	<12 years of education	551 (55.7)	122 (51.3)	429 (57.1)	
	>12 years of education	438 (44.3)	116 (48.7)	322 (42.9)	
Annual Family income ^1^					0.214
	<$35,000	118 (13.0)	34 (15.5)	84 (12.3)	
	>$35,001–$70,000	228 (25.2)	47 (21.4)	181 (26.4)	
	>$70,001	559 (61.8)	139 (63.2)	420 (61.3)	

^1^ Average gross salary in Australia in 2009 was $63, 612 [26]; * Significant at *p* < 0.05.

### 3.2. Types of Supplements Consumed

The most common supplement was a multivitamin, used by 42% of male supplement users and 33% of female supplement users. This was followed by vitamin C, consumed by 39% of male supplement users and 29% of female supplement users. Only two female supplement users consumed a folate supplement. Protein was only taken by male supplement users. No participants were taking a dedicated vitamin D supplement (Table 2).

**Table 2:** Nutritional supplements consumed by 17 year old adolescents in the Western Australian Pregnancy Cohort (Raine) Study.

Supplement Type	Males	Females
	(*n* = 96)	*(n* = 142)
	*n* (%)	
Vitamin C	37 (39.0)	41 (29.0)
Vitamin B/B complex	4 (4.2)	8 (5.6)
Folate	0 (0.0)	2 (1.4)
Iron	4 (4.2)	35 (24.6)
Calcium	1 (1)	7 (5.0)
Magnesium	5 (5.2)	7 (5.0)
Zinc	8 (8.3)	12 (8.5)
Multivitamin/mineral	40 (42.0)	47 (33.0)
Fish/Cod liver oil	25 (26.0)	37 (26.0)
Primrose/Starflower Oil	4 (4.2)	4 (2.8)
Probiotics	2 (2.1)	4 (2.8)
Protein ^1^	7 (7.3)	0 (0.0)
Other ^2^	9 (9.4)	12 (8.5)

^1^ Protein powder, micronized creatine monohydrate, muscle building supplement, lipo 6, mixed amino acids, complete protein; ^2^ Fibre, cranberry, phytelle, garlic and horseradish, echinacea, garlic, glucosamine, chondroitin, spirulina, olive oil extract, butter menthol, l-lysine.

### 3.3. Median Daily Micronutrient Intakes from Food and Supplements

When micronutrient intakes from food only were compared between users and non-users, male supplement users had significantly higher intakes than male non-users (*p* < 0.05) for all nutrients with the exception of vitamin D (Table 3). Female supplement users had significantly higher intakes of magnesium, potassium, vitamin A, beta-carotene, pantothenic acid, pyridoxine, folate and vitamin C (*p* < 0.05) from food sources than female non-users. In supplement users, all micronutrient intakes were significantly higher (*p* < 0.05) from food and supplements compared with food only.

### 3.4. Adequacy of Intakes

Fewer than 50% of females (both supplement non-users and supplement users) met the EAR for calcium, magnesium, folate or the AI for vitamins D and E (Table 4). Fewer than 50% of male non-users met the EAR for magnesium, potassium, pantothenic acid, folate or the AI for vitamins D and E. From food sources only, in male supplement users, fewer than 50% met the EAR for folate or the AI for vitamins D and E. When the contribution of supplements was accounted for, there was an approximate 20% increase in the number of males and females meeting the EAR for folate and those meeting the AI for vitamins D and E. However, more than 50% of males still failed to meet the AI requirements for vitamin D and more than 50% of females failed to meet the requirements for folate, vitamins D and E as well as calcium and magnesium.

## 4. Discussion

We found that adolescents in Western Australia had intakes below recommendations for calcium, folate and vitamins D and E. Females also had low intakes of magnesium. Low micronutrient intakes were also found in other studies carried out in Australia amongst 16–18 years old, where females reported low intakes of calcium, folate, and magnesium, and males reported low intakes of folate, calcium and potassium [1]. Similarly, in the UK, adolescent males aged 11–18 years had low intakes of magnesium, potassium, zinc, folate, iron and vitamin D, while females of the same age had low intakes of calcium, magnesium, potassium, folate, iron and vitamin D [30]. In 11–14 year old adolescents in Spain, more than 50% of males had intakes below the recommended nutrient intake (RNI), for magnesium, calcium, folate and vitamins A, B6, D and E, while more than 50% of females had intakes below the RNI for magnesium, calcium, folate and vitamins A, B6, D and E [31]. In the US around 90% of adolescent girls have inadequate intakes of calcium, magnesium, potassium and vitamins D and E, and many do not meet the recommendations for, zinc, phosphorous and vitamins A, B6, B12 and C [32].

**Table 3:** Median daily micronutrient intakes from food and supplements in male and female supplement non-users (*n* = 753) and users (*n* = 238) in 17 year old adolescents in the Western Australian Pregnancy Cohort (Raine) Study.

Nutrient	Supplement Non-Users (*n* = 753)		Supplement Users (*n* = 238)		Supplement Users (*n* = 238)	
	Food Sources		Food Sources		Food and Supplements	
	Males (*n* = 358)	Females (*n* = 395)	Males (*n* =96)	Females (*n* = 142)	Males (*n* = 96)	Females (*n* = 142)
	Median		Median		Median	
Calcium (mg)	1089.5	842.3	1395.3 *	851.4	1402.1	880.9
Iron (mg)	13.8	10.8	16.4 *	11.1	19.4	14.8
Zinc (mg)	12.8	10	14.6 *	10.1	18.8	11.7
Magnesium (mg)	309.1	248.1	397.8 *	265.7 *	428.2	287.8
Potassium (mg)	3421.8	2890	4232.9 *	3167.7 *	4243.5	3180.1
Phosphorous (mg)	1654.4	1252.9	2064.5 *	1293.8	2064.5	1293.8
Copper (mg)	1.9	1.5	2.2 *	1.7	2.2	1.7
Vitamin A (µg)	1003.1	886.7	1218.9 *	985.2 *	1447.6	1136.7
Beta-carotene (µg)	3229.5	3397.3	3805.4 *	3733.6 *	4113.1	4494.5
Thiamin (mg)	1.8	1.3	2.1 *	1.3	2.7	1.6
Riboflavin (mg)	2.3	1.8	2.9 *	1.9	3.8	2.3
Niacin (mg)	38.6	29.6	44.5 *	29.7	54.3	35.3
Pantothenic acid (mg)	5.2	4.2	6.3 *	4.6 *	8.7	5.4
Pyridoxine (mg)	1.6	1.4	2.0 *	1.5 *	2.9	2.4
B12 (µg)	4.6	3.4	5.1 *	3.4	7.4	4.3
Folate (µg)	252.4	204	313.1 *	224.4 *	399.9	280.8
Vitamin C (mg)	146.4	128.3	178.4 *	142.9 *	335	253.3
Vitamin D (µg)	1.7	1.3	1.9	1.2	3	1.5
Vitamin E (mg)	7.1	5.8	8.6 *	5.9	11.5	6.9

* Significant difference in micronutrient intakes from food sources between supplement users and non-users (*p* < 0.05).

**Table 4:** Number and percentage [*n* (%)] of adolescents meeting the EAR or AI [27] in male and female supplement non-users (*n* = 753) and users (*n* = 238) in 17 year old adolescents in the Western Australian Pregnancy Cohort (Raine) Study.

	EAR/AI		Supplement Non-Users (*n* = 753)		Supplement Users (*n* = 238)		Supplement Users (*n* = 238)	
			Food Sources		Food Sources		Food and Supplements	
Nutrient	Males	Females	Males (*n* = 358)	Females (*n* = 395)	Males (*n* = 96)	Females (*n* = 142)	Males (*n* = 96)	Females (*n* = 142)
			*n* (%)		*n* (%)		*n* (%)	
Calcium (mg) ^1^	1050	1050	185 (51.7)	116 (29.4)	70 (72.9) *	50 (35.2)	73 (76.0)	56 (39.4)
Iron (mg) ^1^	8	8	336 (93.9)	312 (79.0)	92 (95.8)	114 (80.3)	94 (97.9)	128 (90.1)
Zinc (mg) ^1^	11	6	248 (69.3)	354 (89.6)	79 (82.3) *	126 (88.7)	95 (99.1)	128 (90.1)
Magnesium (mg) ^1^	340	300	153 (42.7)	125 (31.6)	59 (61.5) *	58 (40.8) *	75 (78.1)	67 (47.2)
Potassium (mg) ^1^	3600	2600	163 (45.5)	239 (60.5)	63 (65.6) *	101 (71.1) *	85 (88.5)	101 (71.1)
Phosphorous (mg) ^1^	1055	1055	308 (86.0)	266 (67.3)	90 (93.8) *	102 (71.8)	90 (93.8)	102 (71.8)
Copper (mg) ^1^	1.5	1.1	260 (72.6)	327 (82.8)	82 (85.4) *	126 (88.7)	94 (97.9)	128 (90.1)
Vitamin A (µg) ^1^	630	485	296 (82.7)	356 (90.1)	89 (92.7) *	132 (93.0)	95 (99.0)	134 (94.4)
Beta-carotene (µg) ^1^	n/a	n/a						
Thiamin (mg) ^1^	1.1	0.9	332 (92.7)	318 (80.5)	90 (93.8)	125 (88.0) *	94 (97.9)	130 (91.50
Riboflavin (mg) ^1^	1.1	0.9	339 (94.7)	363 (91.9)	93 (96.9)	137 (96.5)	96 (100)	137 (96.5)
Niacin (mg) ^1^	12	11	358 (100)	391 (99.0)	95 (99.0)	139 (97.9)	96 (100)	139 (97.9)
Pantothenic acid (mg) ^1^	6	4	133 (37.2)	214 (54.2)	54 (56.3) *	93 (65.5) *	94 (97.9)	100 (70.4)
Pyridoxine (mg) ^1^	1.1	1	304 (84.9)	306 (77.5)	90 (93.8) *	122 (85.9) *	95 (99.0)	129 (90.8)
B12 (µg) ^1^	2	2	345 (96.4)	332 (84.1)	92 (95.8)	114 (80.3)	95 (99.0)	118 (83.1)
Folate (µg) ^1^	330	330	45 (12.5)	26 (6.5)	94 (26.3) *	46 (32.4) *	63 (65.6)	61 (43.0)
Vitamin C (mg) ^1^	28	28	349 (97.5)	388(98.2)	96 (100)	141 (99.3)	96 (100)	141 (99.3)
Vitamin D (µg) ^2^	5 ^1^	5 ^1^	17 (4.7)	5 (1.3)	11 (11.5) *	3 (2.1)	33 (34.4)	38 (26.8)
Vitamin E (mg) ^2^	10 ^1^	8 ^1^	90 (25.1)	96 (24.3)	56 (58.3) *	33 (23.2)	56 (58.3)	57 (40.1)

EAR, Estimated Average Requirement; AI, Adequate Intake [27]. ^1^ EAR; ^2^ AI; * Significant difference in the percentage of adolescents meeting the EAR or AI from food sources only between supplement users and non-users (*p* < 0.05).

Approximately 24% of adolescents in this cohort consumed supplements. The prevalence of supplement use varies in other countries from 20% to 26% in adolescents in the US [14,33] and 11%–45% in European countries [5,13,14]. In our adolescent cohort, supplement use significantly increased the intakes of all nutrients in both males and females. However, even among supplement users, intakes of some micronutrients, including calcium, folate, vitamins D and E, remained lower than the recommendations. The low intakes of calcium, folate and vitamin D raises concern since inadequate calcium intake may lead to decreased bone-mineral density and increased risk of developing osteoporosis [16], low folate intake in females may lead to neural tube defects in the baby [34], and low vitamin D levels may affect both skeletal and non-skeletal health [35].

Vitamin D occurs naturally in very few foods, many of which are consumed episodically and contain relatively small amounts of vitamin D [36]. The low dietary supply of vitamin D makes it unrealistic that individuals would achieve the recommended intake of vitamin D. Indeed, the major source of vitamin D is exposure to sunlight and dietary sources of vitamin D are not necessary in the presence of adequate sunlight exposure. The AI for vitamin D assumes no, or minimal, sunlight exposure and individuals who do not meet the AI for vitamin D may have sufficient vitamin D status based on exposure to sunlight. Furthermore, care should be taken when estimating inadequacy based on an AI: although the AI can be used as a goal for individual intake, there is limited certainty about the value [27].

Particular characteristics appear to be associated with supplement use. We found that supplement users were more likely to be physically active than non-users. This finding is supported by Reaves *et al*. 2006 in the CATCH study, where 9–18 year old supplement users were more physically active than non-users and 47% of supplement users participated in team sports compared to 40% of non-users [14]. Adolescent supplement users in the NHANES were more physically active and were more likely to engage in less than 2 h of television/video or computer use per day than non-users [15]. In Finland, leisure time physical activity was the strongest factor associated with supplement use in 12–18 year old adolescents [13]. Grm and colleagues [5] showed that 12 and 17 year old adolescent supplement users were more likely to be members of sports clubs than non-users.

Higher supplement use in physically active adolescents may be due to the perceived benefits of dietary supplements on performance [37]. Indeed, protein supplements were more commonly consumed by males in our study and in others [5,13,38], which may be due to the perceived benefits of protein in increasing or improving sports performance [33]. In general, there appears to be a discrepancy in the type of supplements consumed and the micronutrients that are deficient in the diet. For example, almost all adolescents in this study achieved the recommended intake for vitamin C through food alone, even though vitamin C was the most commonly consumed supplement. In contrast calcium intakes were low in females but calcium supplements were only consumed by 3% of supplement users. It has been suggested that manufacturers develop a multivitamin/mineral that responds to the need for micronutrients that may be inadequate in the diet [39].

Supplement users in this study had higher micronutrient intakes from food sources alone compared with non-users. Therefore, supplements were less likely to be consumed by those who would benefit most from them. This pattern was also observed in the National Health and Nutrition Examination Survey (NHANES), where supplement non-users were more likely to have inadequate intakes of calcium, magnesium, phosphorous, vitamins A and C from food sources, than supplement users [40]. Reaves *et al*. 2006 in the Child and Adolescent Trial for Cardiovascular Health (CATCH) study found that adolescents who followed a healthier dietary pattern were also supplement users and those facing the greatest risk of micronutrient deficiencies were less likely to use supplements [15]. This limits the effectiveness of supplementation as a public health strategy to increase micronutrient intakes.

One strategy to improve micronutrient inadequacies is to promote a balanced diet rich in nutrient dense foods. Another alternative to supplementation is fortification of foods with micronutrients known to be low in the population. For example, in order to increase folate intakes at a population level, all wheat flour for making bread (excluding organic) in Australia is fortified with 2–3 mg of folic acid per kilogram of wheat [41]. In the US and Canada, foods are widely fortified with vitamin D in order to increase intakes and reduce to the risk of vitamin D deficiency [42].

## 5. Conclusions

Finding a balance between inadequate and excessive nutrient intakes is paramount to ensuring healthy development in adolescents. Along with increasing the consumption of nutrient-dense foods, supplement use may help to correct micronutrient imbalances. However, our results suggest that those who use supplements have higher micronutrient intakes from food sources and are less likely to require supplements than non-users. Furthermore, the type of supplements used by adolescents may not match the micronutrient deficiencies in the diet. Professional advice should be sought for correcting micronutrient imbalances using food and/or supplements.
